# Age and Sex Differences of Virtual Reality Pain Alleviation Therapeutic During Pediatric Burn Care: A Randomized Clinical Trial

**DOI:** 10.1089/jmxr.2024.0004

**Published:** 2024-07-24

**Authors:** Katerina Jones, Megan Armstrong, John Luna, Rajan K. Thakkar, Renata Fabia, Jonathan I. Groner, Dana Noffsinger, Ai Ni, Bronwyn Griffin, Henry Xiang

**Affiliations:** ^1^ College of Medicine, Northeast Ohio Medical University, Rootstown, Ohio, USA.; ^2^ Center for Pediatric Trauma Research, The Abigail Wexner Research Institute, Nationwide Children’s Hospital, Columbus, Ohio, USA.; ^3^ Center for Injury Research & Policy, The Abigail Wexner Research Institute, Nationwide Children’s Hospital, Columbus, Ohio, USA.; ^4^ IT Research and Innovation, The Abigail Wexner Research Institute, Nationwide Children’s Hospital, Columbus, Ohio, USA.; ^5^ Trauma and Burn Program, Nationwide Children’s Hospital, Columbus, Ohio, USA.; ^6^ College of Medicine, The Ohio State University, Columbus, Ohio, USA.; ^7^ Division of Biostatistics, The Ohio State University College of Public Health, Columbus, Ohio, USA.; ^8^ NHMRC Centre of Research Excellence-Wiser Wound Care, Menzies Health Institute of Queensland, Griffith University Brisbane, Gold Coast, Australia.

**Keywords:** virtual reality, burn, pediatric, pain

## Abstract

Virtual reality (VR) effectively alleviates pain for pediatric patients during many medical procedures, such as venipuncture and burn care. In our previously published randomized clinical trial among 90 pediatric burn patients, participants in the active VR group had significantly lower scores for overall pain compared with participants in the standard care control and for worst pain compared with participants in the passive VR and control group. However, whether VR differs by a patient’s age or sex remains unresolved. Thus, we reanalyzed our data by comparing the active and passive VR participants to evaluate how age and sex affect VR pain alleviation during dressing care for pediatric burns. In total, 90 patients aged 6–17 years (inclusive) with burn injuries were recruited from an outpatient burn clinic of an American Burn Association-verified pediatric burn center. Before randomization, VR helpfulness and need expectations were assessed on a visual analog scale (0–100). Participants were randomly assigned to active VR, passive VR, or control for one dressing change. Immediately following the dressing change, active and passive VR participants self-reported pain and the time spent thinking about pain and rated the VR features on the degree of realism, pleasure/fun, and perceived engagement level. Path analyses assessed how these VR features were interrelated and how they affected self-reported pain by age and sex. Patients aged 6–9 years reported higher mean expectations of VR helpfulness and need (mean = 73.6 and 94.5, respectively) than 10–12-year-olds (mean = 55.7 and 84.2, respectively) and 13–17-year-olds (mean = 68.6 and 77.4, respectively). The path analysis indicated VR engagement and fun were significantly correlated (*p-*value < 0.05). VR engagement significantly negatively impacted overall pain scores (coefficient = −0.45, −0.41; *p-*value < 0.05) and significantly positively impacted time thinking of pain (coefficient = 0.38, 0.32; *p-*value < 0.05). Younger patients had the highest expectations of VR helpfulness and need. VR game realism, fun, and engagement features were not statistically different between age groups and sexes. VR engagement and thinking of pain during burn dressing significantly positively affected self-reported pain (*p-*value < 0.05), suggesting an analgesic mechanism beyond distraction alone. Younger patients benefited more from VR than older patients.

## Introduction

In recent years, several nonpharmacological methods for pain alleviation have been explored, including virtual reality (VR). VR has been established as an effective method to distract pediatric patients and alleviate pain during particularly painful medical procedures.^1^ Although VR has been associated with some short-term side effects because of simulator sickness (including nausea, eye strain, dizziness, and headache),^2^ VR is generally considered a promising nonpharmacological approach for further research and clinical use.^3^ In particular, VR pain alleviation therapeutics (VR-PAT) are very effective among pediatric patients.^4^ For example, VR reduces pain in younger children during burn hydrotherapy sessions.^5^ Although VR has been used for many clinical pain conditions^6–10^ that include wide age ranges and both sexes, whether VR pain alleviation differs according to a patient’s age or sex remains unresolved. Whether and how a patient’s age or sex may influence VR-PAT during medical procedures needs further investigation.

Burn injury is among the top ten leading causes of death and unintentional injury in children.^11^ Over 67,000 US children sustained nonfatal burn injuries in 2020.^12^ Patients with severe burns experience background pain that exists during rest and procedural pain from wound care procedures adds to this existing pain.^13^ Painful burn dressing experiences can stress patients significantly enough to affect postinjury health outcomes.^14^ Pharmacological methods, such as opioids, are often used as the standard treatment to address this pain^13^; however, opioid tolerance and dependence may increase over time.^15^ Owing to the current US opioid abuse epidemic, nonpharmacological approaches to alleviate pain are particularly needed.

Pain is a subjective experience, with pain perception and rating differing based on sex.^16,17^ However, many studies use experimental pain conditions,^18–20^ which are different from pain caused by medical conditions such as burn injury. Around puberty, sex differences in pain emerge, with adolescent girls reporting more pain than adolescent boys.^21^ In addition, pediatric patients tend to process pain in more complex ways as they age.^22^

Our team recently conducted a large between-groups randomized controlled trial (RCT) study of the effectiveness of VR pain alleviation among 6–17 years of pediatric burn patients.^23^ Using between-group *t*-tests, we found that active VR was significantly more effective at reducing worst pain than passive VR. After our publication, we realized that key features of VR and the age and sex of participants might have significantly impacted their rating of VR experience and pain scores. In the current article, we reanalyzed the same dataset to evaluate how age and sex affect VR pain alleviation during painful burn wound cleaning procedures. We aimed to (1) assess whether age or sex impacted how our VR-PAT affected pain during pediatric burn dressings, (2) evaluate whether different perceptions of VR-PAT (game realism, fun, and engagement) correlated with pain alleviation, and (3) evaluate whether prior expectations of VR efficacy significantly influenced pain alleviation during burn dressing changes. Our central hypothesis was that VR features (game realism, fun, and engagement) could be rated differently depending on the age or sex of the patients. In turn, these key VR features could significantly impact the effectiveness of VR-PAT.

The current article uses the same dataset as in the previous publication to answer new questions not addressed before.^23^ The main results presented in the current article are original (not previously published, except the new nonparametric statistic comparison of overall and worst pain scores between active vs. passive VR participants were noted with footnotes).

## Methods

### Data source

Data used in these analyses were collected from our previously published RCT designed to assess VR-PAT efficacy to manage pain during pediatric burn dressing changes in an outpatient clinic. The study design and protocols are described in a prior publication.^23^ Patients were randomly assigned to an active VR group (immersive VR with gameplay), passive VR group (same immersive environment without gameplay), or standard care control group (using conventional distraction methods available in the clinic, such as iPads, music, books, and/or talking). Self-reported, observed, and burn wound data were collected during one scheduled dressing change within a 6-day median since the initial injury at the outpatient burn clinic of an American Burn Association (ABA)-verified pediatric burn center.

The Institutional Review Board of the children’s hospital reviewed and approved the study. Written informed consent from one legal guardian and written assent from children 9 years and older were obtained before beginning study measures. This study was registered at ClinicalTrials.gov, with the identifier NCT04544631.

### Study population

Inclusion criteria were children aged 6–17 years (inclusive) treated at the outpatient burn clinic of an ABA-verified pediatric burn center between December 2016 and January 2019 and spoke English as their primary language. Exclusion criteria were (1) a severe burn on the face or head that prevented VR use; (2) cognitive or motor impairment that prevented study measure administration; (3) visual or hearing impairments that prevented VR interaction; or (4) did not have a legal guardian present to give consent. These inclusion and exclusion criteria (including age range) were designed to be most appropriate for VR immersion and administration of study measures.

### Study procedures

After informed consent but before randomization, a trained researcher conducted a preintervention survey, asking patients about their expectations of VR distraction effectiveness. These questions were “How much would you like to have something fun to do during the dressing change?” and “How much do you think it would help with your pain during the dressing change?” These questions were assessed using a 100 mm visual analog scale (VAS), where a higher number indicated more expectation of effectiveness. Guardians were asked whether the child took any pain medications within 6 h before the burn wound care appointment at the outpatient clinic.

Following the preintervention survey, participants were randomized to active VR, passive VR, or the control group. The randomization scheme used a prospective three-group between subject 1:1:1 allocation ratio, which was and was balanced by sex (male/female) and stored in a Research Electronic Data Capture database.^24,25^

The VR-PAT was hosted on a low-cost VR viewer and displayed on an Apple iPhone 6 (similar to a Google Cardboard set-up). Participants in the active VR group played a VR game entitled “Virtual River Cruise,” which was developed internally at Nationwide Children’s Hospital. Children interact with the immersive VR environment by tilting their head, minimizing potential interference with the dressing change procedure. Participants in the passive VR group were immersed in the same VR environment without the interaction components of the game.

Immediately following the burn wound care, another trained researcher blinded to the patient’s intervention group conducted a postintervention survey. Patients were asked to report their overall pain, worst pain, and time spent thinking about pain during the burn dressing change procedures using the VAS (0–100, with higher scores indicating more pain or more time thinking about pain). Participants in the VR groups were asked to rate the VR game’s realism (“How realistic did you feel about it?”), fun (“How much fun did you have with the VR?”), and engagement (“How engaging did you think it was?”) using the 0–100 VAS, with higher scores indicating more realism, fun, or engagement.

Patient demographic information and burn injury characteristics were obtained from each patient’s electronic medical record. For this analysis, demographic variables included sex (male, female) and date of birth (used to calculate age and grouped as 6–9, 10–12, and 13–17 years). Burn injury characteristics included the percentage of total body surface area (TBSA) (<1%, 1.0–4.9%, 5.0–25.0%) and burn degree (first, second, and third). Nurses involved in burn wound care were asked to report the degree of healing of the wound using a Likert scale (minimal healing present, partially healed burn [<50% healed], mostly healed burn [>50% healed], or completely healed).

### Outcome measures

The primary outcome was a self-reported overall pain score during the burn dressing procedure. After the burn wound care, a trained researcher asked the patients about their overall pain during the whole burn dressing change using a VAS (range 0–100, with higher scores indicating more pain).

### Statistical analysis

Based on published RCT studies,^23,26^ we assumed a medium effect size (*f*^2^ = 0.15) of the active VR. Using two tails and α  =  0.05, we estimated a fixed-effect linear regression model offering power >0.80 with a total sample size of 90 children. We aimed to recruit 30 participants for each group (active VR, passive VR, and standard-of-care control group) to ensure adequate study power.

As this study aimed to determine how overall pain score differed by age and sex, and we postulated features of the core VR experience (VR game realism, fun, and engagement) would vary by age and could significantly impact the effectiveness of VR-PAT, the standard-of-care group was excluded from the final statistical analysis. We first compared the frequency of active versus passive VR assignment, burn injury characteristics (%TBSA, burn degree, and healing degree), and any pain medication used within 6 h before the burn wound care by study participants’ sex and age categories (6–9, 10–12, and 13–17 years) to ensure comparability of patients in active versus passive VR groups. We then compared the numerical overall pain score, VR expectation, and core VR experience (VR game realism, fun, and engagement) using mean, median, lower quartile, and upper quartile by sex and age categories. The *p-*values of the statistical difference test were calculated from nonparametric Wilcoxon rank sum tests.

We conducted path analysis modeling to estimate the hypothesized relationships among key VR features (VR game realism, fun, and engagement) and their impact on the primary outcome (patient self-reported pain score) via the intermediate variable “time thinking about pain” during burn dressing. An extension of multiple regression, path analysis estimates the magnitude and significance of hypothesized connections between variable sets.^27^ Path analysis also allows simultaneous assessment of multiple independent variables’ direct and indirect effects on the primary outcome variable (self-reported overall pain score during burn dressing). A direct effect is observed when an independent variable directly influences a dependent variable. In contrast, an indirect effect occurs when an independent variable affects a dependent variable through a mediating variable. Standardized path coefficients with corresponding *p-*values could help determine independent variables’ significant direct and indirect effects on the study’s primary outcome variable.

We used SAS version 9.4 to conduct data analyses.^28^ Statistical significance was set at α < 0.05, and all tests were two-tailed.

## Results

**FIG. 1.**
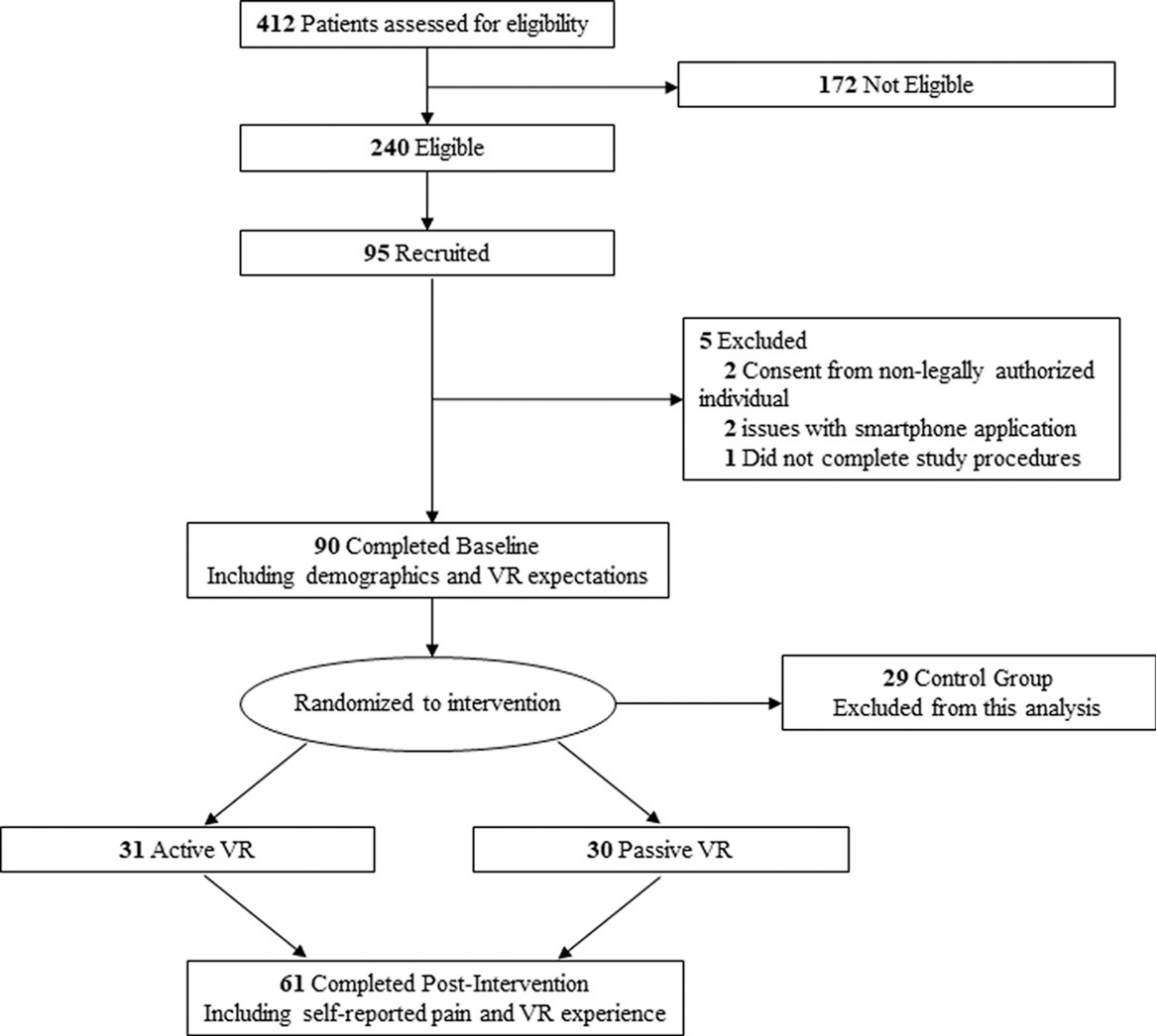
CONSORT flow diagram of patient screening, recruitment, and study procedures.

In total, 412 children were screened for eligibility, with 240 eligible and 95 recruited into the study (Fig. 1). Of those recruited, 90 completed the preintervention survey and were randomized. For this analysis, the 29 patients randomized to the control group were excluded to assess age and sex differences in their VR features rating, self-reported time thinking about pain, and overall pain intensity score during burn dressing care, leaving 61 subjects for the current analysis.

Burn characteristics and pain medication used within 6 h before the burn dressing care by age and sex are reported in Table 1. Age and sex did not significantly differ by the VR group assignment, TBSA burned, burn degree, healing degree, or pain medication within 6 h of dressing (*p*-value < 0.05).

**Table 1.** Demographics and Burn Characteristics of Participants Aged 6–17 Years Who Used VR-PAT During Burn Dressing Changes (*N* = 61)

	Sex			Age category			
	Male (*n*, %)	Female (*n*, %)	*p*	6–9 yrs (*n*, %)	10–12 yrs (*n*, %)	13–17 yrs (*n*, %)	*p*
VR group			0.89				0.90
Active VR	16 (50.0%)	15 (51.7%)		10 (32.3%)	9 (29.0%)	12 (38.7%)	
Passive VR	16 (50.0%)	14 (48.3%)		11 (36.7%)	9 (30.0%)	10 (33.3%)	
TBSA burned			0.31				0.57
Missing	0 (0.0%)	1 (3.5%)		0 (0.0%)	1 (5.6%)	0 (0.0%)	
<1%	12 (37.5%)	11 (37.9%)		6 (28.6%)	6 (33.3%)	11 (50%)	
1.0–4.9 %	15 (46.9%)	16 (55.2%)		13 (61.9%)	9 (50.0%)	9 (40.9%)	
5–25%	5 (15.6%)	1 (3.5%)		2 (9.5%)	2 (11.11%)	2 (9.1%)	
Burn degree			0.09				0.20
Missing	0 (0.0%)	1 (3.5%)		0 (0.0%)	1 (5.6%)	0 (0.0%)	
Second degree	28 (87.5%)	28 (87.5%)		18 (85.7%)	16 (88.9%)	22 (100%)	
Third degree	4 (12.5%)	0 (0.0%)		3 (14.3%)	1 (5.6%)	0 (0.0%)	
Heal degree			0.95				0.52
Minimal	13 (40.6%)	10 (34.5%)		10 (47.6%)	2 (33.3%)	7 (31.8%)	
Partially healed (<50%)	9 (28.1)	8 (27.6%)		6 (28.6%)	4 (22.2%)	7 (31.8%)	
Mostly healed (>50%)	7 (21.9%)	8 (27.6%)		2 (9.5%)	6 (33.3%)	7 (31.8%)	
Completely healed	3 (9.4%)	3 (10.3%)		3 (14.3%)	2 (11.1%)	1 (4.6%)	
Pain medication within 6 h before dressing change			0.30				0.11
Missing	0 (0.0%)	2 (6.9%)		0 (0.0%)	2 (11.1%)	0 (0.0%)	
No	20 (62.5%)	18 (62.1%)		12 (57.1%)	13 (72.2%)	13 (59.1%)	
Yes	12 (37.5%)	9 (31.0%)		9 (42.9%)	3 (16.7%)	9 (40.9%)	

TBSA, total body surface area; VR, virtual reality; VR-PAT, virtual reality pain alleviation therapeutics; yrs, years.

The self-reported experience of 61 patients using VR-PAT is reported in Table 2. VR expectations differed by age and patients who expected VR to be helpful and necessary for pain alleviation. Younger patients 6–9 years had higher expectations of VR helpfulness and pain alleviation needs (mean = 73.6 and 94.5, respectively) than those 10–12 years (mean = 55.7 and 84.2, respectively) and 13–17 years (mean = 68.6 and 77.4, respectively). Females were significantly more likely to rate VR fun (*p*-value = 0.04). However, VR game realism or engagement did not significantly vary by age or sex. In addition, VR expectations or overall pain scores did not significantly differ by sex. However, overall pain scores differed by age, with the youngest patients reporting the highest overall pain (mean = 40.4), compared with those 13–17 years (mean = 16.0; *p-*value = 0.02). These pain results held for self-reported worst pain with the youngest patients reporting the highest worst pain scores (mean = 47.1), compared with those 13–17 years (mean = 20.5; *p-*value = 0.02). Consistent with our previous publication,^23^ self-reported worst pain score was significantly lower in active VR participants in comparison with passive VR participants (*p-*value = 0.04).

**Table 2.** Self-Reported Experience of 6–17-Year-Old Patients Who Used VR-PAT During Burn Dressing Changes (*N* = 61)

Variable	Same size (*n*)	Mean	Median	Lower quartile	Upper quartile	p value^¥^
Overall pain						
Male	32	34.7	9.0	0.0	71.0	0.19
Female	29	25.3	4.0	0.0	50.0	
6–9 yrs	21	40.4	20.0	0.0	76.0	0.02
10–12 yrs	18	35.9	11.5	0.0	64.0	0.06
13–17 yrs	22	16.0	0.0	0.0	45.0	Reference
Active VR	31	24.9	5.0	0.0	50.0	0.23
Passive VR	30	35.8	9.0	0.0	70.0	
Worst pain						
Male	32	39.7	22.5	0.0	79.0	0.48
Female	29	35.1	15.0	0.0	75.0	
6–9 yrs	21	47.1	27.0	0.0	96.0	0.02
10–12 yrs	18	46.9	45.0	7.0	80.0	0.02
13–17 yrs	22	20.5	0.0	0.0	50.0	Reference
Active VR	31	27.4	11.0	0.0	53.0	0.04
Passive VR	30	47.9	50.0	0.0	95.0	
VR expectations – sex						
Male helpful	32	67.3	66.0	50.0	87.5	0.35
Female helpful	29	65.6	60.0	50.0	99.0	
Male need	32	88.2	98.5	80.0	100.0	0.41
Female need	29	82.1	100.0	67.0	100.0	
VR expectations – age						
6–9 yrs helpful	21	73.6	83.0	50.0	100.0	0.34
10–12 yrs helpful	18	55.7	50.0	50.0	65.0	0.09
13–17 yrs helpful	22	68.6	67.5	50.0	90.0	Reference
6–9 yrs need	21	94.5	100.0	95.0	100.0	<0.01
10–12 yrs need	18	84.2	99.0	50.0	100.0	0.23
13–17 yrs need	22	77.4	80.0	67.0	100.0	Reference
VR realism						
Male	31	61.5	74.0	30.0	100.0	0.15
Female	29	71.0	84.0	49.0	100.0	
6–9 yrs	21	74.1	95.0	40.0	100.0	0.22
10–12 yrs	17	57.8	50.0	30.0	100.0	0.42
13–17 yrs	22	64.8	68.0	50.0	95.0	Reference
VR fun						
Male	32	75.2	89.5	55.0	100.0	0.04
Female	29	88.6	99.0	92.0	100.0	
6–9 yrs	21	83.9	100.0	86.0	100.0	0.48
10–12 yrs	18	73.4	90.5	50.0	100.0	0.19
13–17 yrs	22	86.0	98.5	78.0	100.0	Reference
VR engaging						
Male	32	73.1	88.0	62.0	100.0	0.35
Female	29	78.8	99.0	61.0	100.0	
6–9 yrs	21	73.5	91.0	54.0	100.0	0.36
10–12 yrs	18	80.6	87.0	61.0	99.0	0.18
13–17 yrs	22	80.6	100.0	70.0	100.0	Reference

^¥^ One-sided *p-*value from Wilcoxon rank-sum statistic test.

VR, virtual reality; VR-PAT, virtual reality pain alleviation therapeutics; yrs, years.

The standardized direct and indirect effects of age and sex on VR pain scores calculated from path analysis models are reported in Table 3. VR engagement significantly negatively affected pain scores in both models (coefficient = −0.45, −0.41, *p-*value < 0.05, respectively). Time thinking of pain during burn dressing care significantly affected both models (coefficient = 0.38, 0.32, *p-*value < 0.05, respectively), proving a significant role of distraction in pain alleviation. In the path analysis model by patient age, age significantly negatively impacted the self-reported overall pain score (coefficient = −0.30, *p* < 0.05), suggesting that VR pain alleviation decreased as the patient’s age increased.

**Table 3.** Standardized Direct and Indirect Effects^a^ of Sex, Age, and Virtual Reality Features on Self-Reported Overall Pain Score (0–100) During Pediatric Burn Dressing Changes (6–17 Years, *N* = 61)

Effect	Direct	Indirect	Total
Sex (male = 1; female = 0)	0.10	0.03	0.12
VR fun (0–100)	−0.12	−0.06	−0.18
VR engaging (0–100)	−0.39^b^	−0.05	−0.45^b^
Time thinking of pain during burn dressing (0–100)	0.37^b^		0.38^b^
Age (6–17 years)	−0.20^b^	−0.10	−0.30^b^
VR fun (0–100)	−0.17	−0.05	−0.22^b^
VR engaging (0–100)	−0.37^b^	−0.04	−0.4^b^
Time thinking of pain during burn dressing (0–100)	0.32^b^		0.32^b^

^a^ Two separate path analysis models were conducted for age and sex.

^b^ *p* < 0.05.

VR, virtual reality.

**FIG. 2.**
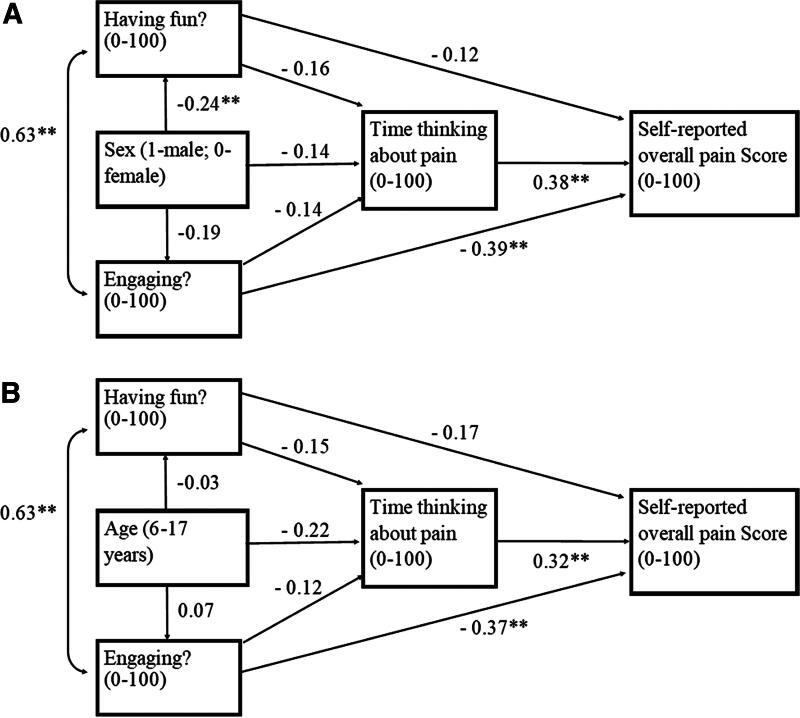
Postulated sex path model **(A)** and age path model **(B)** for VR pain effect during dressing changes. Association between multiple variables and patient self-reported pain is shown by arrows extending from each variable to self-reported overall pain score (0–100). Numbers on each arrow are standardized path coefficients. The higher coefficient indicates a stronger association where **indicates *p* < 0.05. VR, virtual reality.

The association between multiple variables and patient self-reported pain by sex and age is shown in Figure 2A, B. VR engagement and fun were significantly correlated (coefficient = 0.63, *p-*value < 0.05). VR engagement and overall pain score were significantly negatively correlated by sex and age (coefficient = −0.39, −0.37; *p-*value < 0.05). Time spent thinking about pain during burn dressing care had a significant positive correlation with pain score by sex and age (coefficient = 0.38, 0.32; *p-*value < 0.05). The patient’s sex and reporting of having fun were significantly negatively correlated (coefficient = −0.24, *p-*value < 0.06), suggesting that female patients had more fun than male patients.

## Discussion

In our previously published RCT study,^23^ we found the smartphone VR-PAT showed efficacy in reducing observed and patient self-reported pain during burn dressing changes. Patients and their caregivers were satisfied with the VR-PAT, and pediatric patients considered the VR-PAT fun, engaging, and realistic.^23^ After our publication, our team realized that key features of VR and the age and sex of participants might have significantly impacted their rating of VR experience and pain scores, but an in-depth analysis was not done.

The current article investigated how patient expectation of VR-PAT, need for VR, and key VR features (VR game realism, fun, and engagement) could affect the self-reported pain intensity score during pediatric burn dressing care across age ranges and by sex. VR realism and engagement did not significantly vary by sex or age. VR engagement correlated with greater fun and lower overall pain scores across age and sex, whereas time thinking about pain during burn dressing care correlated significantly with increased self-reported overall pain scores. Younger patients expected the VR experience to be more necessary and helpful in reducing pain, and female patients reported more fun with VR than male patients.

Our results suggest nearly all pediatric patients, regardless of age and sex, could benefit from the VR-PAT and experience a significantly reduced overall pain intensity during burn dressing procedures. However, our findings differ from a previous study where younger children had more “fun” with VR.^29^ Future research could include investigations on how “fun” by age differs in VR-PAT.

In our study, VR engagement was significantly correlated with fun, and fun and engagement significantly reduced pain intensity scores in the path analysis model assessing patient age, VR fun and engagement, and time thinking about pain during burn dressing care. Compared with a noninteractive VR experience, interactive and engaging VR was considered more fun,^30^ and VR that is demanding and engages more mental load reduced the intensity and unpleasantness of nociceptive stimuli.^31^ In addition, patients who reported more fun also reported lower pain intensity scores,^32^ which could be because children are generally more responsive to VR-PAT that involve play.^33^ Subjective pain intensity ratings (via functional magnetic brain imaging) significantly decreased during VR immersion, followed by decreased pain-related brain activity and higher inhibition of the pain matrix in the brain.^34,35^ These brain imaging data suggest a neuromechanism for the nonpharmacological analgesia achieved through VR pain alleviation. The presiding theory for how VR alleviates pain is that VR competes for a fixed amount of the user’s attention, replacing nociceptive input with pleasant sensory input through descending inhibitory pathways.^36^ Our study provides further evidence that VR engagement, which correlates with fun, could alleviate pain on a neurological level beyond mere distraction (i.e., time thinking about pain during burn dressing care).

Engaged patients reported lower pain scores and patients who spent more time thinking about pain reported higher pain scores. Pain requires attention,^37^ so the attention-grabbing nature of VR could leave less attention available to process incoming pain signals.^38^ Previous trials have indicated that VR reduces the time patients think about pain.^31,39^ By engaging their attention with the VR-PAT, patients could have spent less time thinking about pain during the burn dressing care and, in return, subjectively experienced less pain.

Younger patients had higher expectations of VR-PAT’s helpfulness and need. Pain expectations can influence how children perceive, express, and respond to pain.^40^ In a previous study of expectations going into a gaming experience, participants with favorable expectations of the distraction reported more enjoyment and engagement and lower pain intensity scores.^41^ The youngest group reported the highest pain intensity scores in our study. However, the age path analysis model suggested that age had a significant, negative direct, and overall impact on self-reported pain intensity. Our findings were consistent with a prior study that examined VR distraction during dental anesthesia, in which the youngest patients reported the highest pain scores.^42^ When compared with video games, head-mounted display VR raised the pain tolerance and threshold in children over ten but did not significantly affect participants under ten,^43^ which could be because of younger children’s higher sensitivity to noxious stimuli.^44^ Overall, age differences in VR-PAT remain largely unresolved.

A strength of our study was that demographics and burn characteristics of our patients were comparable, i.e., the randomized patients did not significantly differ by age, sex, or burns treated. One limitation was that the effect of VR-PAT was only measured once, instead of during repeated burn dressing care over time. However, previous studies found that VR is effective for multiple treatments, including our study using VR-PAT for a week of at-home burn dressing changes.^45–47^ Future research could consider a crossover study design between active and passive VR over multiple burn dressings to account for individual differences in pain during wound care. Another limitation of our study is that VR experience and pain intensity scores were subjective and self-reported by the patients, which is a common weakness in the pain research field. Objective measures such as neuroimaging biomarkers should be developed and tested in future research. Finally, we used a smartphone-based VR as the intervention in this study. Since designing this study, VR technology has increased and become more accessible for medical VR research, which is a consideration for future research studies. For example, we have transferred our Virtual River Cruise game to a Pico Neo Pro Eye 3 headset for future research, allowing for increased graphics and higher immersion into the environment. However, depending on the research question and population of interest, smartphone-based VR could still be a very appropriate option to bridge the digital divide.

## Conclusions

VR was similarly fun, engaging, and realistic across age but less effective in alleviating pain in older children. VR engagement and time spent thinking about pain during burn dressing had a significant direct positive effect on self-reported pain, suggesting an analgesic mechanism beyond distraction alone. Virtually reality is becoming increasingly used in medical settings, so future development and research of VR-PAT should include key VR features that could significantly impact VR pain alleviation effectiveness.

## Abbreviations Used

ABA: American Burn Association
RCT: randomized controlled trial
TBSA: total body surface area
VAS: visual analog scale
VR: Virtual Reality
VR-PAT: VR pain alleviation therapeutics
